# Probing the interaction of lipids with the non-annular binding sites of the potassium channel KcsA by magic-angle spinning NMR

**DOI:** 10.1016/j.bbamem.2011.09.017

**Published:** 2012-01

**Authors:** Phedra Marius, Maurits R.R. de Planque, Philip T.F. Williamson

**Affiliations:** a School of Biological Sciences, Highfield Campus, University of Southampton, Southampton, SO17 1BJ, UK; b School of Electronics and Computer Science, University of Southampton, SO17 1BJ, UK

**Keywords:** Ion channel, Solid-state NMR, Lipid/protein interface, Phosphorous NMR, Magic-angle spinning, Potassium channel KcsA

## Abstract

The activity of the potassium channel KcsA is tightly regulated through the interactions of anionic lipids with high-affinity non-annular lipid binding sites located at the interface between the channel's subunits. Here we present solid-state phosphorous NMR studies that resolve the negatively charged lipid phosphatidylglycerol within the non-annular lipid-binding site. Perturbations in chemical shift observed upon the binding of phosphatidylglycerol are indicative of the interaction of positively charged sidechains within the non-annular binding site and the negatively charged lipid headgroup. Site directed mutagenesis studies have attributed these charge interactions to R64 and R89. Functionally the removal of the positive charges from R64 and R89 appears to act synergistically to reduce the probability of channel opening.

## Graphical abstract

**Figure:**
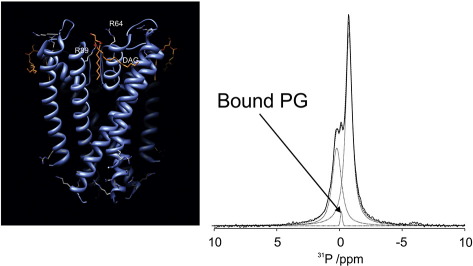

**Highlights:**

► Solid-state 31P MAS NMR studies have been used to characterise binding to the non-annular binding site of KcsA. ► Residues R64 and R89 have been identified as important in the recognition of anionic lipids in the non-annular binding site. ► Single-channel current recordings demonstrate the functional importance of these interactions for KcsA activity.

## 1 Introduction

Interactions of integral membrane proteins with their surrounding lipid environment play a key role in stabilising their structure and influencing their activity. To obtain insight into the nature and type of interactions occurring between lipids and integral membrane proteins a range of biophysical techniques including electron spin resonance [1,2] and fluorescence [3,4] spectroscopy have been used in numerous studies. This has provided a wealth of information on the specificity of proteins for particular classes of lipids and the affinity of these interactions [5,6]. These studies have revealed that in addition to the ‘bulk’ lipids whose dynamic properties remain largely unchanged by the presence of integral membrane proteins within the bilayer, there exist a population of lipids whose motional freedom is constrained through their interaction with integral membrane proteins. The motionally restricted population of lipids can be segregated into ‘annular lipids’ which exhibit low affinity interactions with the hydrophobic surface of membrane proteins and ‘non-annular lipids’ which exert high affinity interactions at sites located in clefts on the protein surface or at the interface between protein subunits [5,7–9]. The interactions at both sites play an important role in modulating the function of integral membrane proteins, but it has recently become apparent that occupation of the non-annular binding site can be particularly critical for function [10]. Despite the importance of these interactions a detailed atomic level description of the type and nature of the interplay between lipids and integral membrane proteins is limited as the lipids are often missing from high-resolution crystal structures of membrane proteins.

One of the best-studied ion channels is the potassium channel, KcsA from *Streptomyces lividans*. This potassium channel, in common with other members of this family, has an absolute requirement for anionic lipids such as phosphatidylglycerol, phosphatidylserine or cardiolipin for function; in their absence the channel exists in a non-conducting state [11,12]. The binding of lipids to KcsA has been extensively investigated by fluorescence quenching studies that clearly identify annular and non-annular populations of lipids [7,10]. Binding of lipids to the annular sites revealed marked differences between the inner and extracellular leaflets, with the extracellular side showing similar affinities for anionic and zwitterionic lipids. In contrast the intracellular side showed a twofold higher affinity for anionic lipids over phosphatidylcholine, presumably due to the clustering of charged residues on KcsA close to the bilayer surface. Fluorescence quenching studies have revealed that the non-annular binding sites show a high degree of selectivity of binding anionic lipids almost exclusively, albeit with moderate affinity.

The crystal structure of KcsA in detergent has provided valuable insights into the interaction of lipids with the non-annular binding site with electron density being seen at the interface between the protein subunits indicating the presence of a diacylglycerol-like moiety. The crystal structure revealed that the *sn*-1 chain of this lipid molecule was tightly buried in the groove between the pore helix and the M2 helix whilst the *sn*-2 chain was less intimately associated with the protein [11,13]. Subsequent studies have revealed the lipid present in the KcsA crystals to be phosphatidylglycerol which co-purifies with the KcsA [11] (pdb accession number 1K4C), suggesting that the phosphatidylglycerol headgroup exhibits significant motion or disorder within the crystal, resulting in the absence of electron density. The absence of electron density in the region corresponding to the lipid headgroup has precluded a detailed understanding of how the phosphatidylglycerol is recognised. The presence of two key arginine residues (R64 and R89) in close proximity to the proposed headgroup region suggested a putative role for electrostatic interactions between the sidechains and the anionic lipids within the binding site [11]. The proposed interactions between R64 and R89 have been investigated by molecular dynamics, revealing the formation of H-bonds between the headgroups of the anionic lipids phosphatidic acid and phosphatidylglycerol and the arginine residues [14]. Notably, these interactions were absent or reduced in bilayers containing the zwitterionic lipid phosphatidylethanolamine [14].

To demonstrate the feasibility of detecting interactions between non-annular binding sites and anionic lipids we have undertaken a 31P magic-angle spinning (MAS) NMR study of KcsA reconstituted into a model lipid bilayer composed of phosphatidylcholine and the negatively charged phosphatidylglycerol. The application of MAS permits the acquisition of ^31^P NMR spectra in which the individual lipid components are resolved on the basis of their chemical shift [15]. The chemical shift observed provides information on the local electrostatic environment of the phosphate moiety of the lipid headgroup and is thus an excellent reporter on the interaction of the lipid headgroup with the protein [16–18]. Typically the application of NMR to study lipid/protein interactions has proved challenging as exchange between the bulk lipid and annular sites occurs too rapidly on the NMR timescale to permit the observation of the bound lipids. Furthermore, interactions between the lipids and the proteins are typically hydrophobic in nature with little difference in electrostatic environment between the bound and free lipids resulting in only minor perturbations in chemical shift. In contrast, phosphatidylglycerol bound at the non-annular binding site is predicted to experience a significantly different electrostatic environment from the annular/bulk lipids with higher-affinity interactions resulting in longer residency times within the binding site. Below we demonstrate that this results in a spectroscopically distinct species that we resolve in the ^31^P MAS-NMR spectrum of KcsA reconstituted into lipid vesicles, providing insights into the interactions involved in lipid binding. Using site directed mutagenesis we have demonstrated that the perturbation in electrostatic environment arises through the interaction of the lipid head group with the positively charged sidechain of R64 and R89. Single-channel current recordings have been measured to ascertain the role that these residues play in determining the channel gating behaviour of KcsA.

## 2 Materials and methods

### 2.1 Materials

Palmitoyloleoyl-phosphatidylcholine (POPC) and palmitoyloleoyl-phosphatidylglycerol (POPG) were purchased from Avanti Polar Lipids (Alabaster, AL). The pQE32 vector and M15[PREP] *Escherichia coli* strain were bought from Qiagen (UK). The detergent, dodecylmaltoside (DDM) was from Anatrace (UK). The other reagents for the purification were obtained from Sigma (UK).

### 2.2 Cloning and mutagenesis of KcsA

The pQE32 vector containing the KcsA gene with a hexahistidine epitope at the N-terminus, kindly donated by Professor Lee (University of Southampton, UK), was expressed in M15 cells. Three KcsA mutants were generated by site-directed mutagenesis using the Quik-change protocol from Stratagene (La Jolla, CA). Three KcsA mutants were prepared replacing the arginine with the slightly smaller but uncharged leucine at residue 64 (R64L), 89 (R89L) or at both sites (R64,89L). The mutants were generated by PCR using synthetic oligonucleotide primers containing the desired mutations (Eurofins MWG, UK). Complementary oligonuceotides, 5′-AGCTGATCAC GTATCCGTTA GCGCTGTGGT GGTCC-3′ and 5′-GACCACCACA GCGCTAACGG ATACGTGATC AGCTG-3′ were used as forward and reverse primers respectively to create the R64L mutation. Similarly, R89L was produced using the synthetic oligonucleotides 5′-GTGACTCTGT GGGGCCTGC TCGTGGCCG TGGTGGTGA T-3′ and 5′-ATCACCACCA CGGCCACGA GCAGGCCCC ACAGAGTCA C-3′ as primers. Polymerase chain reaction was used to generate the mutants and the resultant PCR product was Dpn1- treated for 1 hr at 37 °C to digest any methylated parental DNA. The DNA was used to transform competent M15 [PREP] *E.coli* cells that were then plated onto agar plates supplemented with ampicillin. Mutations were confirmed by sequencing.

### 2.3 Over expression and purification of KcsA

M15 *E.coli* cells transformed with KcsA or one of the KcsA mutants were used to inoculate 10 mL of Luria Broth (LB) medium containing 100 μg/mL of ampicillin. The overnight culture was then used to inoculate 1 L of LB containing 100 μg/mL of ampicillin and grown to an OD_600_ of 0.8 at 37 °C. Over-expression of KcsA wild type and mutant protein was induced by the addition of IPTG to a final concentration of 1 mM and the culture was grown for a further 4 h at 37 °C. The cells were harvested at 4 °C by centrifugation at 12,000 *g* for 20 min. The cell pellet was resuspended in buffer A (50 mM Tris, 150 mM NaCl, 150 mM KCl, pH 7.4) with 1 mM PMSF and sonicated on ice for 5 min: 15 s on; 20 s off at power level 7 (Misonix sonicator). The membrane fraction was clarified by ultracentrifugation at 420,000 *g* for 40 min at 5 °C. The membrane-containing pellet was homogenised and then gently stirred in solubilisation buffer (buffer A, 40 mM imidazole, 1 mM DDM, pH 7.4) for 1 h at room temperature. Insoluble material was removed by centrifugation at 21,000 *g* for 20 min at 5 °C and the supernatant was loaded onto a Ni^2+^ affinity column (GE Healthcare, UK) pre-equilibrated with buffer A. The column was washed with 20 bed volumes of wash buffer (Buffer A, 40 mM imidazole, 1 mM DDM, and pH 7.4) and KcsA was eluted in Buffer A, 400 mM imidazole, 1 mM DDM, pH 7.4).

### 2.4 Reconstitution of KcsA

KcsA wild type and mutants were reconstituted in lipid vesicles composed of POPC and POPG at a molar ratio of 70:30 respectively. Briefly, 10 mg of lipids in chloroform were dried in a vacuum desiccator and resuspended in hydration buffer (10 mM Tris, 100 mM KCl, 1 mM EDTA pH 7.4) containing 40 mM octyl glucoside. Following mixing and sonication to clarity, KcsA protein was gently added and mixed to give a lipid to KcsA tetramer molar ratio of 100:1. The vesicles were generated by detergent removal using Bio-beads. For NMR studies the vesicles were pelleted by ultracentrifugation at 100,000 *g* for 30 min at 5 °C and loaded into an MAS rotor for analysis by NMR.

### 2.5 Static and magic angle spinning solid-state ^31^P NMR studies

All ^31^P NMR measurements were performed on a 300 MHz Infinity+ spectrometer (Varian, USA) at a 121.37 MHz using a 4 mm triple resonance MAS probe (Varian, USA). ^31^P NMR spectra were acquired with a 5 μs π/2 pulse for excitation, 70 kHz continuous wave proton decoupling during acquisition and a recycle delay of 3 s. All spectra were obtained at 25 °C with a magic angle spinning frequency of 6 kHz. All spectra were externally referenced to phosphoric acid (85%) with a chemical shift of 0 ppm. Typically magic-angle spinning (MAS) and static NMR spectra were obtained by averaging 4096 transients or 8192 transients respectively and 5 Hz linebroadening was added prior to Fourier Transform. Data processing and analysis were performed in matNMR [19].

### 2.6 Single channel electrophysiology of wild-type, R64L, R89L and R64L/R89L in planar lipid bilayers

Single-channel current recordings were acquired as detailed previously by Marius et al.[10]. Briefly, POPC/POPG at a molar ratio of 70 to 30 was first dried from chloroform and then dissolved in decane to a concentration of 20 mg/mL. A lipid planar bilayer was painted across a 150 μm aperture in a Delrin cuvette (Warner Instruments, CT, USA) which separated two chambers with a volume of 1 mL. The *cis* (extracellular) chamber contained buffer B (10 mM Hepes, 150 mM KCl) at pH 7.0 whilst the *trans* buffer contained buffer B at pH 4.0. Bilayer formation was verified with capacitance measurements. The reconstituted vesicle suspension (5 μL) was added to the *cis* chamber and the transmembrane current was measured using Ag|AgCl electrodes. The electrode in the *cis* chamber was connected to the input of the headstage of an ID562 bilayer amplifier (Industrial Developments Bangor, Bangor, UK), whilst the bias voltage was applied to the *trans* chamber, as described fully in Marius et al. [10]. The bilayer was voltage-clamped at + 100 mV and electrical recordings were digitised at 5 kHz, and subsequently digitally low-pass filtered at 1 kHz for analysis and at 2 kHz for display. Current traces were analysed using Origin (OriginLab, US). Episodes of rapid channel gating, with a duration of 15 s or more, were used for analysis of the channel open probability.

## 3 Results and discussion

### 3.1 Expression, purification and reconstitution of wild type and mutant KcsA

**Fig. 1:**
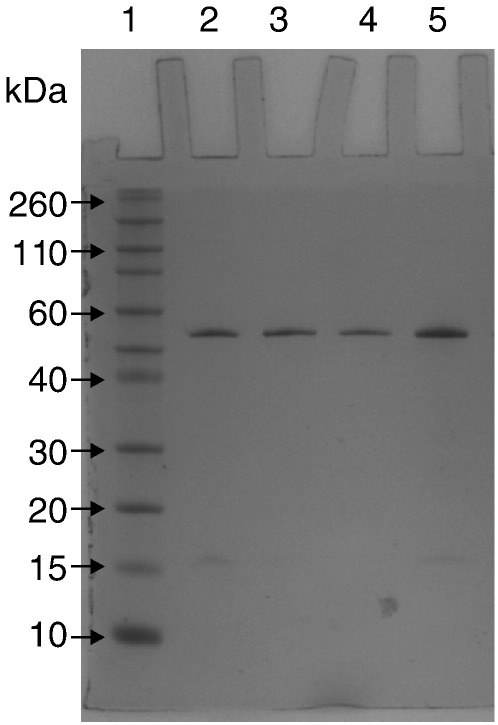
Coomassie stained tricine SDS-PAGE of purified wild-type KcsA (Lane 2) the mutants R64L (Lane 3), R89L (Lane 4), and the R64L/R89L double mutant (Lane 5). Molecular weight markers (Lane 1).

The generation of the KcsA mutants, R64L, R89L and R64, 89L was confirmed by sequencing and mass spectrometry (results not shown). The overexpression of wild type and mutants of KcsA as His-tag fusion protein in M15 cells yielded cell pellets of 3 ± 1 g/L culture. The extraction of the protein from cells involved solubilisation of the membrane fraction using the non-ionic detergent DDM, followed by Ni-NTA affinity chromatography. Increasing the imidazole concentration from 40 mM to 400 mM resulted in the displacement of bound protein. The purity of the proteins was determined by SDS-PAGE (Fig. 1) with the wild type and three mutants running as a stable SDS resistant tetramer. The yield of pure wild type and single mutant KcsA protein ranged from 5 mg to 10 mg/L whilst the double mutant, R64, 89L, had a yield of 4 ± 2 mg/L. Wild type and mutants of KcsA were prepared for solid-state NMR studies by reconstitution into lipid vesicles as described. The proteins were incorporated into pre-formed lipid-detergent micelles. The addition of Bio-beads for the removal of detergent led to the formation of a cloudy suspension of proteoliposomes that were pelleted for subsequent analysis by NMR.

### 3.2 Phosphorous static and MAS NMR of wild type-KcsA reconstituted into PC/PG vesicles

**Fig. 2:**
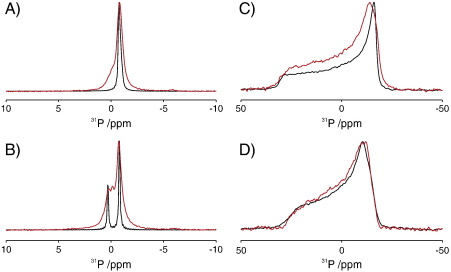
Effect of KcsA on the ^31^P proton-decoupled spectra of lipid vesicles composed of 100% POPC (A/C) and 70% POPC/30% POPG (mol/mol) (B/D) under 6 kHz MAS (A/B) and static (C/D). Spectra of lipid vesicles alone (black) and in the presence of KcsA at a lipid to KcsA tetramer ratio of 100:1 (red).

To ascertain the effect of KcsA on POPC and POPC/POPG (70%/30% mol/mol) vesicles ^31^P solid-state NMR spectra were recorded in the presence and absence of KcsA (Fig. 2). To confirm that the KcsA spectra had been successfully reconstituted into lipid bilayers and that this had not altered the phase behaviour of the lipids, static ^31^P proton decouple NMR spectra were acquired (Fig. 2C and D). In each case the spectra showed the axially symmetric powder pattern expected for POPC and POPC/POPG bilayers in the L_α_-phase, with no evidence of smaller isotropic micellar contributions. Upon the introduction of acidic POPG into the vesicles a significant reduction in the chemical shielding anisotropy was observed, falling from − 31.9 ppm in pure POPC vesicles to − 21.3 ppm in vesicles composed of 70% POPC/30% POPG (mol/mol). Such a reduction has previously been observed and attributed to the change in the surface charge of the bilayer [17,18]. Comparison of the static spectra of pure lipid vesicles and those containing KcsA reveals a similar reduction in chemical shielding anisotropy in both samples indicating that the introduction of KcsA did not significantly perturb the dynamics observed in the bilayer and had no effect on the overall charge on the bilayer surface [17,18].

The MAS spectra of POPC and POPC/POPG (70%/30% mol/mol) of lipid vesicles in the absence of KcsA are shown in Fig. 2A and B (black). The spectra of POPC alone show a single resonance at − 0.8 ppm, whilst the mixture of POPC/POPG shows two well-resolved resonances at − 0.79 ppm and 0.30 ppm, which are assigned to POPC and POPG respectively on the basis of their relative intensities and earlier studies [17,20].

**Fig. 3:**
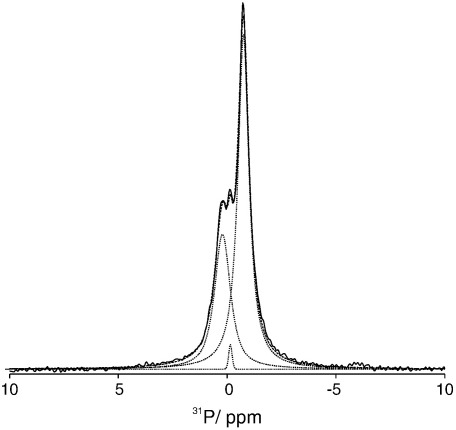
Deconvolution of the ^31^P proton decouple MAS-NMR spectrum of wild type KcsA reconstituted into 70% POPC/30% POPG (mol/mol) bilayer (experimental spectra, solid line; fit of individual components, dotted line; sum of fitted spectral components, dashed line).

The ^31^P MAS-NMR spectrum of KcsA reconstituted into POPC bilayers (Fig. 2A, red) also shows a single resonance at − 0.81 ppm although the linewidth is broader than that for lipid alone, increasing from 0.34 ppm to 0.66 ppm. Such an observation is consistent with earlier studies that also detected an increase in linewidths upon the reconstitution of proteins into lipid bilayers, a behaviour that was attributed to the overall reduction of mobility of the lipids within the bilayer arising from the low lipid to protein ratio. The ^31^P MAS-NMR spectrum of KcsA reconstituted into negatively charged POPC/POPG vesicles (70%/30% mol/mol) is shown in Fig. 2B (red) and deconvoluted into its components in Fig. 3. As expected, two resonances are observed at − 0.75 ppm and 0.23 ppm, but in addition a resonance is also present at − 0.14 ppm. The two larger components at − 0.75 ppm and 0.23 ppm, we assign to the POPC and the POPG present in the bulk lipids respectively. Both resonances show significant broadening compared to the pure lipid vesicles with the linewidths of POPC and POPG increasing from 0.15 ppm to 0.61 and 0.80 ppm respectively. We attribute the presence of the third resonance at − 0.14 ppm to a population of lipid that is bound to the KcsA and therefore observes a different electrostatic environment to that seen by the bulk lipids. Deconvolution of the three spectral components (Fig. 3) indicates that this third component comprises 6.6% of the overall intensity in the ^31^P NMR spectrum and is accompanied by a slight reduction in bulk POPG intensity. On the basis of this reduction in intensity and the known presence of a POPG binding site, we assign this resonance to POPG bound at the non-annular binding site on KcsA. The observed intensity (6.6% of total phosphorous signal) for this bound component is in good agreement with the predicted intensity, which we expect to be maximally 4% of the total intensity when all four non-annular binding sites are occupied with phospholipid and the lipid/KcsA tetramer ratio is 100:1. We note however that with a POPC/POPG ratio of 70%/30% mol/mol some of the binding sites may be occupied with POPC. The appearance of this third resonance and its relatively narrow linewidth (0.21 ppm) when compared to the bulk lipids indicates that there is little exchange on the NMR timescale (microseconds) between the non-annular binding site and the annular/bulk lipid which would otherwise lead to a broadening of the resonance. Comparison of the direct excitation spectra (Fig. 2A/B) with ^1^H-^31^P cross-polarisation (data not shown) showed no significant differences in the relative intensities of the resonances, in agreement with crystallographic studies, indicating that the phosphate does not undergo a significant reduction in mobility upon binding to the non-annular binding site.

The perturbation of the POPG chemical shift upon binding to the KcsA (− 0.37 ppm) indicates that upon binding the phosphate in the lipid headgroup experiences a different local electrostatic environment arising through either changes in local headgroup geometry or local environment. Although a number of factors contribute to the overall chemical shift the upfield perturbation observed is consistent with the interaction of the phosphate group with positively charged residues within the non-annular binding site. Similar upfield perturbations have previously been observed upon the binding of negatively charged lipid species to positively charged binding sites on peripheral membrane proteins [16,21].

### 3.3 Phosphorous NMR of R64L, R89L and R64L/R89L KcsA mutants reconstituted into PC/PG vesicles

**Fig. 4:**
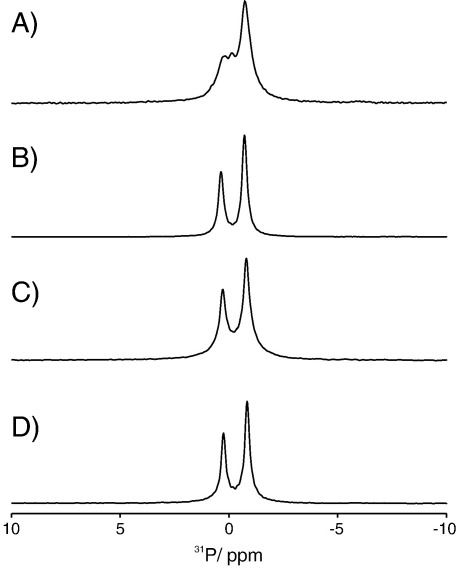
Comparison of ^31^P proton decoupled MAS-NMR spectra of WT KcsA (A) and the site directed mutants R64L (B), R89L (C) R64L/R89L (D) reconstituted into lipid vesicles composed of POPC and POPG at a ratio of 70%/30% (mol/mol).

On the basis of the crystal structure [11] and molecular dynamics simulations [14] of KcsA it had been proposed that R64 and R89 might contribute positive charge to the non-annular lipid binding site (Fig. 6). To ascertain the role of residues R64 and R89 in the binding of POPG to the non-annular binding site, we mutated these residues from arginine to leucine giving three different mutant proteins, R64L, R89L and the double mutant R64L/R89L. The ^31^P proton decoupled MAS-NMR spectra of R64L, R89L and the double mutant R64L/R89L are shown in Fig. 4. The spectra of R64L, R89L and the double mutant R64L/R89L all show two distinct resonances at − 0.75 and 0.23 ppm, corresponding to the POPC and POPG in the bulk lipid respectively. Notably the resonance at − 0.14 ppm that we have attributed to the POPG bound to the non-annular binding site is absent in the spectra of the three mutants. The disappearance of this resonance indicates that the population of lipids previously identified within the non-annular binding site no longer experiences a significant change in electrostatic environment upon binding, either through absence of the positive charges in the binding site or the abolition of lipid binding to these sites. This demonstrates experimentally the observations made in earlier molecular dynamics simulations [14] that indicate both R64 and R89 residues are in close proximity to the phosphate headgroup of the POPG within the non-annular binding site and are implicated in binding either through electrostatic or hydrogen bonding interactions.

### 3.4 Single-channel current recordings of wild-type and mutant KcsA

**Fig. 5:**
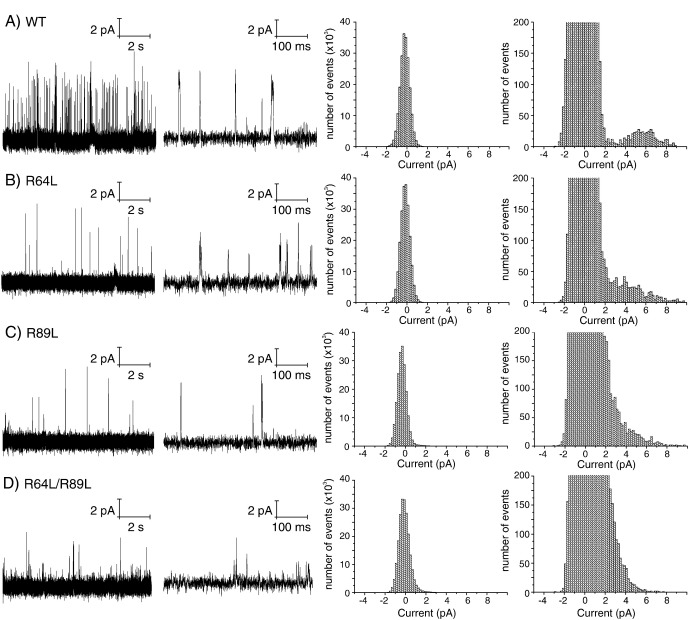
Single-channel recordings from wild-type, R64L, R89L and the R64L/R89L double mutant KcsA showing a reduced channel conductance upon the removal of the positive charge from the non-annular lipid-binding site. Columns 1 and 2: representative single channel recordings, 10 s and 500 ms total time respectively. Column 3: all-point histograms of the open probability of KcsA mutants during a burst of channel activity. Column 4: vertical expansion of column 3, highlighting channel openings.

**Fig. 6:**
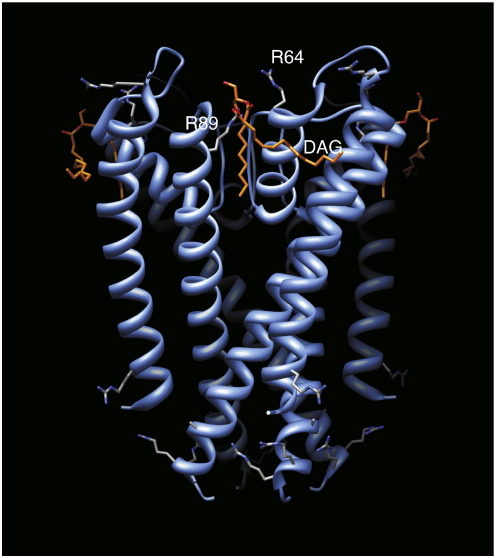
Crystal structure of wild type KcsA showing the location of the resolved DAG buried at the interface between the two subunits. Residues R64 and R89 are both in close proximity to the glycerol backbone of the DAG and well positioned to interact with the negatively charged phosphate group of the anionic lipids which although not resolved in this structure are known to occupy the non-annular binding site. Figure generated from pdb file 1K4C.pdb [26] and visualised in Chimera [27].

To ascertain whether interactions between these positive charge sidechains in the non-annular lipid-binding site and the lipid headgroup could affect channel activity, single-channel current recordings were taken of KcsA wild type and mutants reconstituted in a planar bilayer composed of 70% POPC and 30% POPG (Fig. 5). The bilayer was painted across a 150 μm aperture separating a *cis* chamber, equivalent to the extracellular side of the KcsA channel containing pH 7 buffer, from the *trans* chamber (pH 4) representing the intracellular or cytoplasmic side of the channel. In this configuration, KcsA channels oriented with their protonation sites on the *trans* side will be functional [22]. At 100 mV holding potential, pure-lipid bilayer traces displayed a peak-to-peak noise of ~ 2–4 pA (data not shown). In the presence of wild-type KcsA, the single-channel traces (Fig. 5A) revealed short bursts of activity followed by long periods of inactivity leading to low overall open probability as reported previously [10,23]. Histogram analysis of the activity bursts gave an open probability for the wild-type KcsA of 4.8% with open current amplitudes distributed around 6 pA in agreement with earlier studies [10,23].

The single-channel recordings of the R64L and R89L mutants (Fig. 5B and C respectively) displayed fewer channel openings, however bursts of channel activity were still observed. Using a current amplitude of 4 pA as a threshold, an open probability (P_0_) of 1.6% and 2.0% was measured for the R64L and R89L mutants respectively. In contrast to the wild-type behaviour however, a distribution of current amplitudes is observed ranging from < 4 pA (closed channels) up to a maximum of 10 pA in both cases. We are unable from this data to determine whether the apparent disappearance of a well-defined conductance state is due to channel opening events that are occurring on timescales faster or comparable to our sampling rates or due to an inherent property of the mutant channels, e.g. the existence of additional sub-conductance states. Analysis of the single channel recordings of the R64L/R89L double mutant (Fig. 5D) showed a further decrease in channel opening, with an open probability (P_0_) of 0.8%. As for the single mutants discrete conductance states were not observed.

Despite the open probability of the wild type KcsA being low, the single-channel current recordings obtained for the R64L, R89L and R64L/R89L double mutant clearly indicate that the replacement of the charged arginine residues at position 64 and 89 with leucine alters the gating of KcsA, reducing both its open probability and conductance. Care must be taken in the interpretation of the electrophysiology recordings as lipid protein interactions have been reported to play a significant role in the folding and stability of the tetrameric channel which would clearly have implication on the conductance behaviour [28]. However, the appearance of stable tetramers on the SDS-PAGE of the wild-type and mutants (Fig. 1) suggests that the mutant proteins are both correctly folded and assembled into stable tetramer and thus the changes observed are a result in different gating behaviour. Several patch-clamping studies have previously been reported on the R64A mutant of KcsA reconstituted by mixing bacterial membranes with asolectin vesicles [24,25]. There are significant differences in the responses of the R64A mutation between these studies with Perozo and co-workers reporting a significantly higher open probability of 65% [24] whilst Kremer *et al.* find that the R64A mutant has a higher tendency to close than wild type KcsA [25]. Interestingly, channel-gating behaviour was also reported to be dependent on the nature of the substitution with a R64D mutation giving rise to a higher open probability than R64A, which Kremer et al. suggested to arise from the formation of a salt bridge across the non-annular lipid binding site which may stabilise the open state thereby mimicking the presence of an anionic lipid [25]. The low open probabilities, compared to wild type KcsA, that we observe in the R64L, R89L and R64L/R89L double mutant would be consistent with such a hypothesis, suggesting that the bulky, uncharged sidechains are unable to form the necessary interactions necessary to stabilise the open state.

## 4 Conclusion

Utilising ^31^P proton-decoupled MAS-NMR we have studied the interaction between KcsA and its surrounding lipid bilayer. No specific interaction is observed between KcsA and the zwitterionic POPC membrane constituent, however upon reconstitution into a lipid bilayer composed of POPC/POPG (70%/30% mol/mol), we observe the appearance of a distinct spectral component at − 0.14 ppm that we have assigned to the binding of POPG to the non-annular lipid-binding site on KcsA. The upfield perturbation in chemical shift observed upon the binding of POPG to the non-annular lipid-binding site on KcsA is consistent with the interaction of the anionic lipid with positive charges within the binding site. Based on earlier computational studies and crystallographic studies that proposed a role for R64 and R89 in the recognition of the negatively charged sites in the lipid headgroup we mutated R64 and R89 to a slightly smaller leucine residue. Subsequent ^31^P proton-decoupled MAS-NMR studies of the R64L, R89L and the R64L/R89L double mutant showed the disappearance of the resonance attributed to the bound POPG at − 0.14 ppm. Single channel recordings have revealed that the positive charges on the side chains of R64 and R89 act synergistically to further reduce the low channel open probability observed for KcsA under these conditions. Finally we note that the slow exchange between the lipids at the non-annular binding site compared to the faster exchange observed for annular lipids results in the presence of a distinct spectral component as opposed to broadening. This observation demonstrates the feasibility of observing lipids whilst bound to non-annular binding sites in other, potentially less well characterised systems, providing an alternative biophysical technique to study how lipids and other lipophilic molecules may interact with these sites and the role this plays in regulating the function of integral membrane proteins. Furthermore, the detection of non-annular lipids by NMR paves the way to more complex magic-angle spinning experiments which will enable the accurate structural characterisation of the lipid within its binding site and the interactions responsible for its binding.
